# What Is Trained During Food Go/No-Go Training? A Review Focusing on Mechanisms and a Research Agenda

**DOI:** 10.1007/s40429-017-0131-5

**Published:** 2017-02-22

**Authors:** Harm Veling, Natalia S. Lawrence, Zhang Chen, Guido M. van Koningsbruggen, Rob W. Holland

**Affiliations:** 1 Behavioural Science Institute, Radboud University Nijmegen, Montessorilaan 3, 6525 HR Nijmegen, The Netherlands; 2 School of Psychology, College of Life and Environmental Sciences, University of Exeter, Exeter, UK; 3 Department of Communication Science, Vrije Universiteit Amsterdam, Amsterdam, The Netherlands; 4 Department of Psychology, University of Amsterdam, Amsterdam, The Netherlands

**Keywords:** Go/no-go, Food choice, Impulsive, Training, Eating behavior, Inhibition

## Abstract

**Purpose of Review:**

During food go/no-go training, people consistently withhold responses toward no-go food items. We discuss how food go/no-go training may change people’s behavior toward no-go food items by comparing three accounts: (a) the training strengthens ‘top-down’ inhibitory control over food-related responses, (b) the training creates automatic ‘bottom-up’ associations between no-go food items and stopping responses, and (c) the training leads to devaluation of no-go food items.

**Recent Findings:**

Go/no-go training can reduce intake of food and choices for food and facilitate short-term weight loss. It appears unlikely that food go/no-go training strengthens top-down inhibitory control. There is some evidence suggesting the training could create automatic stop associations. There is strong evidence suggesting go/no-go training reduces evaluations of no-go food items.

**Summary:**

Food go/no-go training can change behavior toward food and evaluation of food items. To advance knowledge, more research is needed on the underlying mechanisms of the training, the role of attention during go/no-go training, and on when effects generalize to untrained food items.

## Introduction

Dietary choices have a profound influence on people’s health and are famously resistant to change. Many people have the intention to eat healthily by limiting their consumption of energy-dense food and about 42% of adults worldwide report trying to lose weight [1]. Yet, the obesity epidemic suggests many people have great difficulty to behave in line with their healthy eating intentions [2]. One explanation for this difficulty is that eating behavior is largely shaped by basic learning mechanisms that reinforce consumption of energy-dense food (e.g., [3]). Specifically, consuming food items containing relatively high amounts of intrinsically rewarding ingredients such as sugar and fat create strong links between these foods and consumption through instrumental or Pavlovian conditioning [4–6]. Furthermore, by learning the rewarding quality of energy-dense food, these foods may subsequently trigger attention and motor responses toward the food, which may further increase the likelihood of consumption, especially when such foods are easily accessible (for a review see [7]). From this perspective, it seems important to consider whether it is possible to change people’s behavior toward energy-dense foods by changing their learned associations with these foods.

Indeed, there is recent interest in changing people’s associations with food items to ultimately change people’s eating behavior. While different approaches exist (see, e.g., [5, 8–10]), we will focus on food go/no-go training [11–13], as this training has been shown to influence various outcome measures such as food choice (e.g., [14]), self-selected portion size [15], consumption volume (e.g., [11]), and body weight ( [12, 16••]; for a review, see [7]). However, it is not yet clear how go/no-go training influences these outcome measures. Therefore, we will review recent findings and discuss to what degree three prominent accounts can explain the effects of go/no-go training in influencing responses to food. We will end by pointing to unanswered questions and outlining an agenda for future research.

## What Is Food Go/No-go Training?

During food go/no-go training, visual (e.g., the border of a picture turning bold [16••]) or auditory go or no-go cues (e.g., a low tone [17••]) are presented in close temporal proximity to images of food items. Participants are instructed to press a button when a go cue is presented and to not press a button when a no-go cue is presented. Importantly, during the training, some food images are consistently presented with no-go cues (no-go items) and other food images or non-food images are consistently presented with go cues (go items). We refer to this training as food go/no-go training. Important features of this training include (a) no-go responses are frequent within the task (usually 50% of the time), (b) there is a high consistency between cue presentation and image presentation (often 100%), and (c) the task is quite easy to perform because the cue is presented at the beginning of a trial and there is ample time to respond (often 1000 or 1500 ms; e.g., [11]). There are also stop-signal variants of this training procedure, but these variants are, as we will briefly discuss afterward, crucially different on a number of features (e.g., [18–20]). The control condition consists of a go/no-go training with non-food images (e.g., [21]) or a (go/no-go) task in which they respond to food images (e.g., [11]).

## A Short Summary of Findings

Go/no-go training can reduce intake of no-go food items compared to conditions in which participants respond to food items, especially among participants scoring high on dietary restraint [11–13, 22]. One experiment showed that go/no-go training compared to a non-food go/no-go training reduced snack intake among children (aged 7–10 years old [21]). Two studies examining the effect of the training on food choice rather than intake found that it reduced choices for no-go food items compared to go food items for participants who were relatively hungry [14, 23] (but see [24]). Another study showed that participants served themselves less no-go sweets compared to responded-to sweets [15]. Furthermore, two studies examined the effects of repeated training on objectively measured weight loss across several weeks. One study found that go/no-go training facilitated weight loss compared to a non-food go/no-go training, especially among participants with a relatively high BMI [25••]. Another study found a main effect of go/no-go training within an overall overweight/obese sample [16••]. Both these studies did not include long-term follow-ups of objectively measured weight. Furthermore, several studies found that the same training procedure can reduce intake of alcoholic beverages [26–29], but in the present review, we will primarily focus on food.

## Possible Mechanisms

How can we explain the nature of the effectiveness of go/no-go training? It has been suggested that repeatedly not responding to specific food items during food go/no-go training (a) trains ‘top-down’ inhibitory control over food-related responses [11], (b) creates direct food item-stop associations, a form of ‘bottom-up’ or automatic inhibition (e.g., [30, 31]), or (c) reduces evaluations of the food items [14, 17••, 23]. Next, we will consider whether these mechanisms operate during go/no-go training, and whether they may explain the behavioral effects.

### Training Inhibitory Control

To what degree are the above-mentioned findings consistent with the idea that food go/no-go training strengthens top-down inhibitory control over food-related responses (e.g., [11])? One difficulty with answering this question empirically is that none of the above studies actually measured inhibitory control as a dependent variable, e.g., by examining the stop-signal reaction time to no-go foods in a different (stop-signal) task. Studies have reported an improvement in response inhibition to no-go foods over the course of training, as measured by fewer commission errors [12], but this could also result from the development of ‘automatic’ inhibition (described subsequently).

We argue that it is highly unlikely that food go/no-go training increases inhibitory control over food-related responses. First, it is important to point out that during food go/no-go training participants hardly make any errors (accuracy tends to be around 95–99%; e.g., [13, 25••]). The task appears very easy to perform, presumably because the response window is not very tight (e.g., 1500 ms), and specific food images are usually paired with no-go cues 100% of the time (for an exception see [12]). It is therefore questionable how such an easy task can train inhibitory control.

Second, research employing a training that actually is considered more demanding for top-down control, the so-called stop-signal training (SST), seems to result in less strong effects compared to those of the go/no-go training (for a meta-analysis see [32]). Specifically, in the SST, participants are presented with items on screen and are asked to make some kind of classification response and refrain from responding when a stop signal (e.g., an auditory cue) is presented. Importantly, the auditory cue is only presented on a minority of the trials (i.e., 25% of the time; cf. go/no-go, 50% of the time) and the timing of the cue is dynamically adjusted using a staircase procedure (i.e., the cue is presented later or earlier during a trial depending on participants’ performance) so that participants manage to stop their responses on time approximately in 50–75% of the trials. The SST is argued to place demands on ‘action cancelation’ after a prepotent response has been initiated rather than the ‘action restraint’ elicited in the go/no-go task [33, 34]. Consequently, the SST may be considered much more of an inhibitory control training compared to go/no-go training, because the SST requires participants to maximize their ability to stop a response toward a food when this is required (e.g., a stop signal is presented).

Interestingly, there are a number of studies that have employed the SST to try to reduce participants’ intake of no-go foods [19, 20], choices for no-go foods [10], or to facilitate weight loss [35]. However, the results are mixed. There are some positive findings (e.g., [20]), but others have found inconsistent [35] or null effects [10, 19], particularly when more demanding SSTs are used which elicit lower rates of successful stopping. Three meta-analyses have shown that the effects of the SST on reducing responses to food items (and alcoholic beverages) tend to be weaker than the effects of go/no-go training (with mean effect sizes of *d* = 0.23–0.26 for SST vs. *d* = 0.47–0.5 for go/no-go [32, 36, 37]).

It has been proposed that the SST may be less effective than go/no-go training because participants respond to the no-go food items in 25–50% of the trials on which they appear (i.e., because they fail to stop on time [32]), leading to less actual inhibition training and hence less effectiveness. Furthermore, it is also possible that the SST encourages learning to stop toward the stop cue rather than to the food compared to go/no-go training, and because the stop cue is not present in real life, the training may become less effective (see [31]). Finally, SST may increase attention and arousal to the no-go food items, because participants need to prepare themselves to quickly stop their responses once they see the no-go item [10]. As a result, the SST may train people inadvertently to attend more to no-go items. Because training attention to food items is known to increase choices for these foods (e.g., [10, 38, 39]), SST may become ineffective under conditions where behavior is strongly guided by attention such as when choosing food [40].

To summarize, the SST, a task considered to train top-down inhibitory control, appears to be less effective than the go/no-go training. This, in turn, makes it unlikely that the go/no-go training effects on behavior are caused by increasing top-down inhibitory control over food-related responses.

### Learning Food-Stop Associations

An alternative explanation that may account for the reduction of responses to food items after go/no-go training is the automatic inhibition account [31]. According to this account, consistently withholding responses toward food items can create a link between these items and stopping [41, 42]. Once such associations have been learned, perception of the items will automatically put behavior on hold [30], perhaps via automatic activation of a ‘stop center’ (as has been proposed for the right inferior frontal gyrus [43]) or by rapid (<100 ms) suppression of motor cortex excitability [44]. This means that during go/no-go training top-down inhibitory control may initially be recruited to withhold responses to no-go stimuli (analogous to a ‘prepared reflex’ to exercise top-down inhibitory control [31]), but recruitment of inhibitory control will decrease over time and becomes superfluous once direct stimulus-stop associations have been acquired, resulting in no-go foods triggering motor inhibition in a stimulus-driven bottom-up manner, analogous to a ‘learned reflex’ [31].

It seems possible that the go/no-go training may create associations between specific food items and stopping a response, although this has not been examined much to date (see [45] for a study on measuring semantic associations between words related to stopping and no-go items after go/no-go training). As mentioned above, several studies have shown that food no-go training leads to improved response inhibition as measured by fewer no-go commission errors to trained items. Improvements are seen both over the course of training and for 100% no-go foods relative to 50% no-go control items [7, 16••], consistent with the notion of an associatively-mediated inhibition response. However, fewer commission errors could result from both improved top-down inhibitory control and from automatic inhibition, making it difficult to disentangle these two mechanisms.

Instead, evidence for the development of ‘automatic inhibition’ comes from studies that show a slowing of reaction times to respond to trained no-go items when these are presented on go trials (in ‘catch’ trials or during test blocks when participants are instructed they will never have to stop to these items). Under these conditions, there is no longer a requirement to inhibit responding to the former no-go items (therefore, improved top-down inhibitory control is irrelevant), but a behavioral effect of training is observed in terms of unintentional slowing of go reaction times. Studies involving food-associated or neutral stimuli have indeed shown such slowed ‘go’ responses to trained no-go items, or to targets presented immediately after them, consistent with the development of automatic inhibition [13, 31, 41, 42, 44]. However, such slowing may only be seen when attention to these task-irrelevant (but no-go-associated) images is increased during training. This can be achieved by presenting the task-irrelevant images shortly (e.g., 100 ms) before the no-go cue, by providing explicit instructions that encourage attending to the images [41, 42] or by using naturally salient (attention-grabbing) images, such as highly palatable foods or cues associated with them [44].

In summary, it seems plausible that stimulus-stop associations develop during food go/no-go training, but their contribution to training effects is unknown.

### Food Devaluation

A third account for the effectiveness of food go/no-go training is offered by the Behavior Stimulus Interaction (BSI) theory [17••, 46] that proposes that appetitive stimuli trigger strong approach reactions that will be inhibited when a no-go cue is presented. Furthermore, to prevent continuous oscillation between approach triggered by an appetitive food item and inhibition of this response, the appetitive item is devalued. By devaluing the food, the approach tendency is reduced and the inhibition toward the food can safely be released in order to select a new course of action. The BSI theory predicts devaluation only for appetitive (food) items and not for neutral items, as neutral items do not elicit approach reactions. This prediction stands in contrast with the devaluation-by-inhibition account that predicts devaluation through inhibition also for neutral stimuli [47, 48]. Thus, according to the BSI theory, reduction of responses to food items after go/no-go training is caused by a reduction in the appetitive value of the no-go items.

A recent series of preregistered experiments indeed found results consistent with those of the BSI theory. Specifically, by assessing evaluations of food items using visual analog scales before and after a go/no-go training, a consistent reduction in evaluations of no-go food items was observed compared to both go food items and food items that were not presented during the go/no-go task [17••]. Furthermore, this devaluation effect was, as predicted, only observed for highly attractive foods, as measured for each participant individually, and not observed for less attractive foods. Moreover, the devaluation effect was visible when go trials were frequent (50 or 75% of the trials), but diminished when go trials became rare (on 25% of the trials). This latter finding suggests that not responding to food items is not sufficient to cause devaluation when this is the default response, but devaluation appears to be caused by ‘active’ response inhibition. Another study suggests the incentive value of no-go food items is decreased by go/no-go training: participants invested less effort to obtain no-go food items compared to go food items (i.e., as indicated by reduced button pressing to obtain no-go food items [15]; for related findings using erotic images, see [49]).

Few studies examined whether devaluation of no-go food items can explain more distal behavioral effects. One study showed that reduced choices for no-go food items compared to go items in a hypothetical choice task was mediated by decreased evaluations of no-go items [14, 23]. Another study found that devaluation of food items by go/no-go training was related to weight loss, but devaluation did not mediate the effect of the training [12, 16••]. Two studies examining go/no-go training using alcoholic beverages as items found that devaluation of no-go beverages as measured with an implicit association test (IAT) mediated subsequent decreased consumption of the beverages [28, 29], but a recent meta-analysis suggests these IAT effects may not be reliable [32] and there is no evidence for this effect in the food domain [22, 24].

To conclude, we think there is strong evidence that no-go food items are devalued (as assessed with explicit questionnaires [12, 14, 16••, 17••, 23]), but the evidence that this is the mechanism underlying effects of go/no-go training on more distal measures such as food intake, food choice, or weight loss is not satisfactory. Nevertheless, of the three accounts, food devaluation seems the best-supported account currently. However, it should be noted that food devaluation is the only mechanism that has been repeatedly examined in food go/no-go training studies. This is probably because assessing food evaluation is quick and simple and does not involve giving participants a task where they have to respond rapidly to no-go foods (i.e., to measure automatic inhibition/slowing to foods), which would counteract the inhibition responses acquired during training and be undesirable in studies with longer-term outcomes.

### Interacting Mechanisms?

Finally, it should be noted that the above mechanisms are not mutually exclusive and may well interact in food go/no-go training or operate at different times. For example, it is possible that early on during training top-down control networks are activated during conflict monitoring, proactive response suppression, and action selection as new food-response contingencies are learned [31]. After a more extensive training, the strength of stimulus-stop associations should be sufficient to cause a more automatic bottom-up form of inhibition based on memory retrieval of stored instances [50]. The motor suppression that follows exposure to trained no-go stimuli [44] may lead to reduced striatal activation (dopamine transmission), consistent with findings that the ventral striatum integrates reward value and motor effort associated with stimuli [51, 52]. This, in turn, could lower the ratings of food liking in evaluation tests and decrease activation in neural regions associated with attention and reward processes [7].

Alternatively, according to the BSI theory, the devaluation of no-go items could occur very early in the learning process, i.e., as soon as conflict between approach and inhibitory responses is detected. In this case, devaluation would not be related to or follow on from suppression of motor cortex excitability but rather would precede it. In support of this, one study suggested that devaluation of no-go foods occurred after as few as four pairings with the no-go cue/response [14, 23]. Interestingly, both the suppression of motor cortex excitability and stimulus devaluation have only been observed for appetitive no-go items and not neutral ones, suggesting some potential commonality between mechanisms.

## Research Agenda

It is clear that more research is needed to examine how food go/no-go training changes behavior toward food. Future studies should examine the interrelationships and temporal sequence of the mechanisms during food go/no-go training to clarify how training ‘cold’ motor inhibition translates into reduced ‘hot’ affective evaluations of reward value. This is not only of theoretical interest but is also much needed to optimize training protocols to obtain long-term behavioral change.

First, if the training works by training inhibitory control over food-related responses, which we think is unlikely (see also [53]), stronger training effects may be created by developing challenging and adaptive tasks that train and strengthen top-down inhibitory control toward food. Alternatively, if the training works via food devaluation, as we propose, more insight is needed on what we exactly mean by devaluation (e.g., devaluation of incentive value or liking) and how devaluation effects can be maintained over time. One possibility is that response inhibition toward food items involves a negative affect (e.g., because response inhibition may be an inherently negative affective signal; e.g., [47]; see also [54, 55]) that becomes associated with the food items [17••, 46]. According to this logic, food inhibition training is not so much a training of some sort but resembles a propositional, affective, or associative learning task. This would imply training may be optimized by enhancing learning and memory consolidation (this would also be beneficial for the learned food-stop association account) or by adding positive and negative reinforcement or an evaluative conditioning component [8, 54].

A second interesting question is whether increased attention to the food images during the training may facilitate learning to associate the food images with response inhibition [41], and whether this, in turn, will lead to stronger behavioral effects. Some work suggests that making the contingencies between images and type of responses very explicit, either by informing participants of these prior to training [56] or by instructing participants to react to healthy food and not to unhealthy food [18], may work well to reduce responses to no-go items. Evidence suggests that implicit and explicit learning can occur simultaneously and combining the two mechanisms may result in the strongest effects of inhibition training [57].

A third important question is to what degree effects of go/no-go training generalize to untrained food items. Although this issue has not been systematically examined, there are some indications that generalization may depend on characteristics of the training. Specifically, Chen and colleagues [17••] employed a go/no-go training in which a variety of food items were used on both go and no-go trials. They found that no-go items were devalued compared to both go items and untrained items. This would suggest a lack of generalization to untrained food items. However, another study employed go/no-go training using jelly candy as no-go items and cute animals as go items. This study observed decreased intake of the trained food (jelly candy) and an untrained food (M&Ms) to the same degree [21]. Similarly, a recent study showed that food no-go (relative to go) training reduced intake of both trained and untrained unhealthy foods in a buffet taste test [22]. Although it is difficult to compare these studies directly, these findings suggest generalization to untrained food items depends on the nature of the go items. When similar food items appear on go and no-go trials, generalization may be low because the food-response associations can only be learned on the item level, whereas if only foods [21] or a distinct subcategory (e.g., ‘unhealthy’ foods [22]) are no-go items, generalization may be more likely because people may learn that these foods in general are no-go items. Studies are needed to test generalization effects systematically.

## Conclusions

To conclude, much still needs to be learned about how food go/no-go training changes behavior toward food. Some studies show promising effects, but there appears to be a lack of knowledge on the underlying mechanisms. To advance the field, we need to perform well-powered studies, including preregistrations and direct replications, and examine the mechanisms of food go/no-go training to be able to determine the theoretical underpinnings of the training and to optimize future applied training protocols.
